# Supplementary material for: Diverse infection models demonstrate robust resistance of Mycobacterium tuberculosis to innate immunity
Source: PLoS Pathog. 2026 Oct 21;22(10):e1014713. doi: 10.1371/journal.ppat.1014713 (PMC13705603; doi:10.1371/journal.ppat.1014713)
Supplement: Supplementary file 1 [file ppat.1014713.s010.pdf]

Response to Reviewers' Comments:

Reviewer #1 Summary:

In this manuscript, Fairgrieve et al. examine whether innate immune mechanisms can restrict Mycobacterium tuberculosis (Mtb) during early infection. Using ultra-low dose aerosol infection, environmental immune priming, and lung-restricted inflammation induced by Legionella pneumophila, the authors conclude that Mtb is intrinsically resistant to innate immune clearance. The study is technically sound and addresses an important, long-standing question in tuberculosis immunology. However, the conclusions rely almost entirely on CFU-based measurements and, in doing so, under interpret several observations that point to altered containment and disease tolerance mechanisms.

Major Comment:

Innate immune priming, dissemination, and disease tolerance require tissue-level analysis:
The central conclusion that innate immunity has a limited capacity to control Mtb is primarily based on pulmonary CFU measurements. While this interpretation is technically correct, it is overly restrictive. It does not fully account for an important result in the study: priming innate immunity with Legionella pneumophila significantly reduces extrapulmonary dissemination of Mtb to the spleen, despite no detectable change in lung CFUs. Reduced dissemination is a biologically meaningful phenotype and should not be considered secondary. Rather, it suggests that innate immune priming alters mechanisms of bacterial containment instead of directly enhancing bacterial killing.

We appreciate the reviewer's support and recognition that this study is technically sound and addresses important questions in the field of tuberculosis immunology. We agree that our manuscript focused almost entirely on innate immune mechanisms of resistance to infection (i.e. control of bacterial CFU) and neglected the question of whether the innate immune system had effects on tolerance (disease symptoms). One challenge we faced is that because we sought to address the question of whether innate immunity contributes to tuberculosis control, we focused on an early timepoint after infection (day 14). We chose this timepoint because it is prior to the onset of the adaptive immune response. Analysis after day 14 would be complicated by a possible contribution of adaptive responses to any phenotype, which would confound our ability to discern the contribution of innate responses. The issue is that at day 14 there are essentially no disease symptoms to follow in the model: weight loss, histopathology, inflammatory cytokines, etc. are all absent or negligible at day 14 (as is discussed more below). Therefore, it is difficult for us to comment on how our data address the role of innate immunity in disease tolerance. However, the reviewer makes an excellent point about dissemination, and we agree that the effect we see on dissemination is indeed a very interesting observation. We have better highlighted this point in the manuscript and have additionally included an additional discussion of disease tolerance (lines 405-416 of the revised manuscript).

This interpretation is further supported by the CD64-positive enriched scRNA-seq analysis, which identifies an increased representation of CD86-positive myeloid cells following Legionella priming. These cells are not classically associated with direct antimicrobial activity. Instead, they clearly show scRNA-seq gene signatures linked to regulation of immune responses, resolution of inflammation, wound healing, regulation of vascular development, lipid and cholesterol handling, including APOE-associated pathways, and metabolic homeostasis, such as responses to insulin signaling. Taken together, these transcriptional features are consistent with a shift toward disease tolerance, in which host tissues limit damage and spatially constrain infection, rather than with enhanced bactericidal activity (references 1-2).

We agree that our scRNA-seq data suggests that Lp-primed mice displayed enrichment of pathways associated with resolution of inflammation and wound healing. We have added analysis and discussion of this point to the manuscript (lines 384-394).

Despite these indications, the manuscript does not experimentally address whether innate immune priming alters lung tissue architecture, inflammatory damage, immune organization, or routes of dissemination. As a result, the conclusion that innate immunity plays only a limited role in controlling Mtb risks being overly CFU-centric and conceptually incomplete.

As discussed above, there is little inflammatory damage or histological changes at the day 14 timepoint. Nevertheless, to confirm whether this is indeed the case, we performed histological analysis (by a blinded trained pathologist) on unprimed or Lp-primed mice infected with Mtb. We have summarized the results of this analysis in the table below. At this early time point, there is limited inflammatory damage to the lung, e.g., there are no granulomas or other characteristic TB lesions. The minor histological changes we observe are not different between Lp-primed and unprimed mice.

Sample	Inflammatory cell aggregation, interalveolar and septal, lung	Inflammatory cell infiltration, perivascular and peribronchiolar, lung	Vasculitis, lymphohistiocytic
Naïve	-	-	-
Lp-primed Sample 1	-	Mild, multifocal, multilobe	Suspected, focal
Lp-primed Sample 2	Minimal (extension from perivascular infiltrate)	Mild, multifocal, multilobe	-
Lp-primed Sample 3	Minimal, focal	Minimal, multifocal, multilobe	Suspected, multifocal
Unprimed Sample 1	Mild, multifocal, multilobe	Mild, multifocal, multilobe	Mild, multifocal
Unprimed Sample 2	Mild, multifocal, multilobe	Mild, multifocal, multilobe	Suspected, focal
Unprimed Sample 3	Mild, multifocal, multilobe	Mild, multifocal, multilobe	Mild, multilobe

Naïve

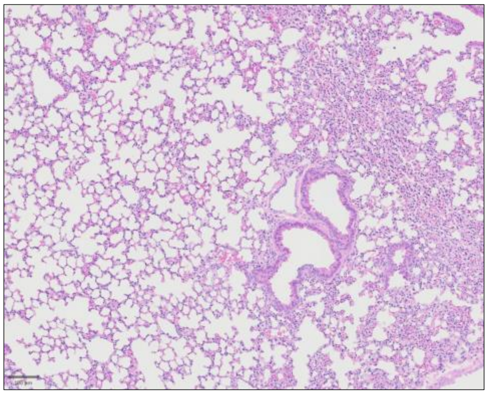

Unprimed

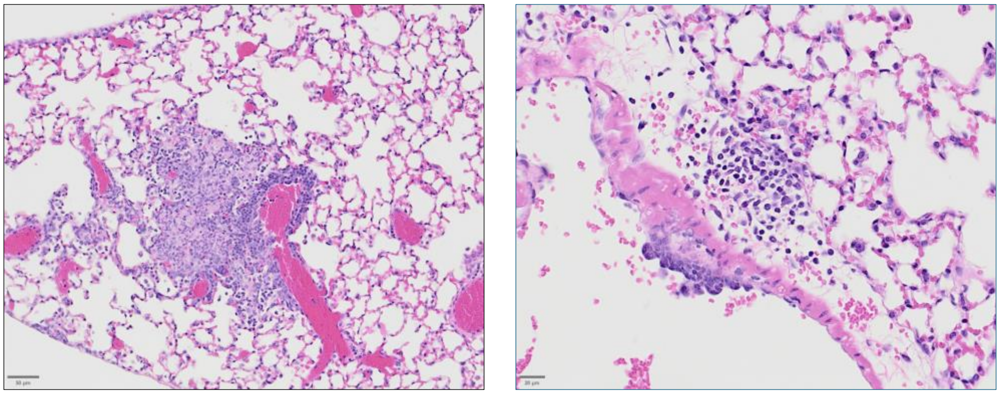

Lp-primed

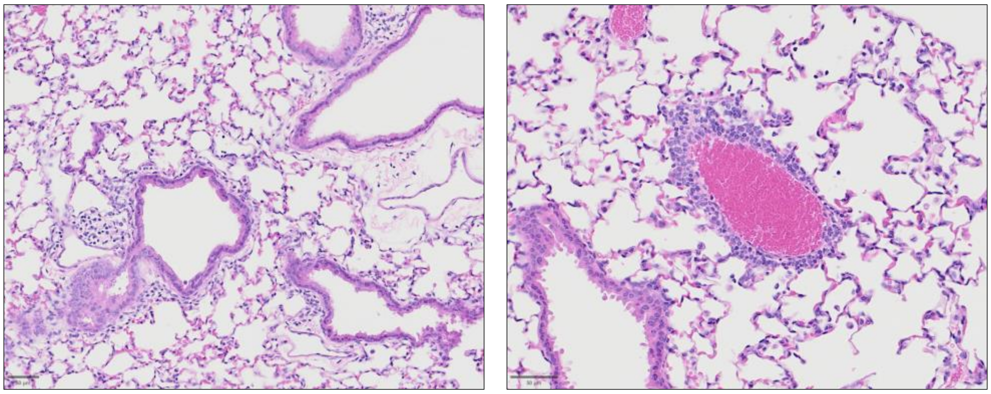

Bibliography:

1. Ayres, J.S., Schneider, D.S. Tolerance of infections. (2012) Annual Review of Immunology. 30:271-94. DOI: 10.1146/annurev-immunol-020711-075030.

2. Medzhitov R, Schneider DS, Soares MP. Disease tolerance as a defense strategy. Science. 2012 Feb 24;335(6071):936-41. doi: 10.1126/science.1214935. PMID: 22363001; PMCID: PMC3564547.

These are terrific papers that we now cite in our discussion of disease tolerance (lines 405-416) of the revised manuscript.

Reviewer #2 Summary:

In this manuscript, Fairgrieve et al. investigates a central and intriguing question in TB immunology: whether pre-existing or infection-induced innate immune activation can control Mycobacterium tuberculosis (Mtb). Prior literature has been showing that innate immunity was less efficient to control Mtb infection, but the findings have not been properly summarized. The authors here provided a summary of the failed control of Mtb replication by innate immunity, by reporting that while innate immune activation controls Listeria or Lp, it fails to restrict Mtb growth. The question is significant, but the study's ambitious conclusion—that the innate immune system cannot restrict Mtb—needs future support. The authors failed to go much beyond CFU measure or to provide mechanistic insights. This lack of deeper investigation limits the novelty and impact. Overall, the conclusion needs more evidence to support. It would be interesting to investigate what is the "innate immunity" activated by ultra-low dose or regular does Mtb? I am also wondering: why does this form of innate immunity fail to restrict Mtb? Does another type of innate immunity exist to restrict Mtb replication?

We appreciate the reviewer's shared interest in the intriguing role of innate immunity in control of Mtb infection. The reviewer's comments somewhat mirror those of reviewer 1, who asked us to consider broader roles of the innate immune system beyond restriction of Mtb replication (CFU levels). We concur with this basic point. While we believe that the analysis of CFU is the correct way to address the central question of our manuscript—does the innate immune system restrict Mtb replication in the lung?—we have, as discussed above, made changes and additions to the manuscript to also discuss broader functional roles of innate immunity in the response to Mtb. We have used other methods, including new experiments that address histopathology, cytokine production, and dissemination of bacteria. It should also be noted that our manuscript employs numerous additional approaches beyond CFU counts, including flow cytometry and single cell RNA-seq, to determine if priming or pre-activating the innate immune system can induce other types of innate immunity that could restrict Mtb replication. The important result of these experiments is that they did not reveal other mechanisms of innate immunity, even the under sensitized conditions we investigate (e.g., ultra-low dose infections, previous priming, etc.). Because we do not find much evidence for Mtb restriction, there isn't much to investigate at greater mechanistic death.

The reviewer asks: "Why doesn't [innate immunity] restrict Mtb?". This is indeed the central question that arises from our work. We do discuss this question in our manuscript (lines 417-431) but given that the answer is likely to be multifactorial, it is unlikely to have a single simple answer that could be addressed in a single manuscript. We consider the significance of our paper to be that we carefully and systemically document the phenomenon of intrinsic resistance of Mtb to innate immunity (using a

variety of approaches that had not previously been attempted to address the question), and we hope that it inspires future work to address the underlying mechanisms.

The reviewer also asks: “Does another type of innate immunity exist to restrict Mtb replication?”. The answer—as discussed in the manuscript and shown previously by numerous other groups—is yes, *adaptive* immunity plays an important role in restricting Mtb replication. However, even adaptive immunity often fails to eliminate the infection. Even though this has been noted for decades, there has never been a clear explanation for the failure of adaptive immunity. However, since adaptive immunity depends on innate mechanisms of killing, our results raise the possibility that the failure of innate immunity might be central to understanding the limited control mediated by adaptive immunity as well.

Reviewer #3 Summary:

The study presented here using different mouse models and set up wishes to address the role of the innate immune defense in the lung against Mycobacterium tuberculosis (Mtb) infection. The authors wish to challenge the concept that local inflammation raised by Mtb infection itself or by co-housing with pet-shop mice or prior controlled infection with another lung pathogen (here Legionella pneumophila/Lp) that activates innate cells -before the onset of adaptive immunity- should be able to eradicate or clear Mtb. The main findings and claims are that primed innate cells in vivo in the lung do no control Mtb multiplication as no difference in CFUs are measured between SPF wild type mice (as commonly used by many other laboratories) and all other mice used (knock-out , co-housed, Lp infected etc...).
There is a consensus that some exposed individuals might be able to resist Mtb infection and deciphering the mechanisms involved is a very important issue that may help to better understand Mtb infection pathophysiology and epidemiology. In that respect the study is timely and important. However, I am not convinced that the methods and data presented really shed new light on the role of innate immunity in Mtb control.

We appreciate the reviewer’s feedback; however, we disagree that our study does not shed new light on the role of innate immunity in Mtb control. While other papers have suggested that innate immunity does not control Mtb replication, these experiments do not consider the effects of infectious dose or prior immune system activation. Indeed, the important contribution of our manuscript is that it carefully assesses whether these factors may have previously resulted in the failure to uncover mechanisms of Mtb control of replication. Our conclusion, which we think is important and perhaps even central to understanding the mystery of Mtb, is that even under sensitized conditions (ultra-low dose, prior innate priming) Mtb exhibits robust resistance to innate control.

Part II – Major Issues: Key Experiments Required for Acceptance
Please use this section to detail the key new experiments or modifications of existing experiments that should be absolutely required to validate study conclusions.

Generally, there should be no more than 3 such required experiments or major modifications for a "Major Revision" recommendation. If more than 3 experiments are necessary to validate the study conclusions, then you are encouraged to recommend "Reject".

Reviewer #1: To support a more comprehensive interpretation, the authors should include tissue-based and inflammatory analyses that are well within the scope of the current study. These could include:

1) Histological analysis:
-H&E staining to assess lung inflammation, tissue damage, and lesion organization, including granuloma aggregates.
- Spatial localization of Mtb within lung compartments using acid-fast staining or fluorescence-based approaches, where applicable.
- Immunohistochemistry or immunofluorescence to evaluate myeloid and lymphoid organization associated with containment.

We performed H&E staining and acid-fast staining on naïve or unprimed/Lp-primed Mtb-infected samples. The samples were analyzed by a blinded expert pathologist. An important point (also discussed above) is that the histopathology at the day 14 is very limited. In addition, acid fast staining revealed the presence of very few bacteria, as is also expected at this early timepoint. Nevertheless, as summarized in the table below, we do not see major differences in pathology between naive and Lp-primed mice. We have included this table and some sample images in Supplementary Figure 5.

2) Measurement of inflammatory mediators in lung homogenates or bronchoalveolar lavage fluid (if available), such as IL-1beta, TNF, IL-6, CXCL1, CXCL2, CCL2, and IL-10.

We measured inflammatory markers in lung homogenates by LEGENDPlex and have included these data in the manuscript (Supplementary Figure 4). We observe only small differences between Lp-primed and unprimed mice at the 14 day time point. As a positive control, we included Mtb-infected mice at 28 days post-infection and observed significantly increased cytokine abundance. Our conclusion is that prior priming with Lp does not dramatically change the inflammatory outcome of Mtb infection.

3) Targeted qPCR analyses from Trizol-preserved lung tissue to assess inflammatory mediators (Il1b, Tnf, Il6), type I interferon and interferon-stimulated gene programs (Ifnb1, Isg15, Mx1), macrophage activation or tolerance markers (Nos2, Arg1, Apoe),

and markers of lung injury or dysfunction (Hmgb1, Mmp9, Mmp12, Sftpc).

As an alternative to this requested experiment, we examined the suggested markers in our single cell RNA-seq data and reported their expression in the manuscript. We observe that the levels of these markers was generally similar between unprimed and Lp-primed samples and several of these markers were not expressed at high levels in either group. However, Lp-primed mice expressed higher levels of *Apoe* and *Mmp12*, while unprimed mice expressed higher levels of inflammatory genes such as *Il1b*, *Isg15*, and *Mx1*. Our conclusion is that prior priming with Lp does not dramatically change the inflammatory outcome of Mtb infection.

Reviewer #2: 1. The ultra-low dose model: The conclusion on that key innate immunity genes known to control Mtb control do not exert substantial effects during the innate phase of the infection, even at highly limited infectious doses is important. The main observation is that the CFU of Mtb was not changed after ultra-low dose infection in all immune deficient mice models. Mice with deletion in Ifngr1, TNFR, Sp140, Myd88/Trif, or harboring Atg5 myeloid deficiency all showed comparable bacterial load at 14 dpi after ultra-low dose Mtb challenge, similar as observed in standard low-dose infection (as cited by the authors and PMID: 37319285). Since the unrestricted control of bacilli growth in innate cells has been reported, the data presented serve to confirm, rather than advance, established knowledge with a newer model. In the standard low-dose model, the failure to control Mtb CFU in these immune deficient mice was observed later at the onset of T cell response. How about in ultra-low dose model?

We conducted a limited number of ultra-low dose infection experiments in which we examined CFU burdens at timepoints after the onset of the T cell response. These experiments were consistent with the expected susceptibility of the innate immune gene knockout mice; however, we did not perform enough replicates to include these data in the manuscript. Again, the focus of our manuscript was on the role of the innate immune system, so we focused on the day 14 timepoint (after which the onset of T cell immunity confounds our ability to attribute any phenotypes to the innate immune system). We agree that it could be interesting to address the role of the adaptive immune system in the ultra-low dose model, but this was not the question we sought to address in our paper (indeed, other labs such as the Urdahl Lab are actively engaged in this work).

Innate immunity has many facets. Directly restriction bacterial replication is only one of it. Therefore, unchanged CFU may not reflect a complete failure of innate immunity.

We agree with the reviewer that the innate immune response can affect pathology (tolerance) during infection in addition to restricting bacterial replication. As described above, we have done additional experiments to examine pathology in the mice, but at the day 14 timepoint, there is in general little pathology and we did not see major effects. However, the reviewers point is well-taken and we have made revisions to the manuscript to be more clear that innate immunity could influence tolerance to the infection in addition to affecting resistance (CFU levels) (lines 405-416 of the revised manuscript).

The innate immunity perhaps is slow in restricting Mtb. 14 dpi CFU provided a snapshot. Not controlling at this timepoint doesn't mean it fails to control infection. It is possible that the "Mtb restriction" is happening in a way undetectable by log changes of CFU.

We chose the day 14 timepoint because it is the latest timepoint at which we can be confident there is not yet any adaptive immune response that would confound our ability to assess the role of innate immunity (again, the clear focus of our study is innate immunity, not adaptive immunity). We agree that there is likely some control of the infection at later timepoints, but we have no way to determine whether this control is due to adaptive immunity (and indeed, much prior literature suggests that it is). We do not understand the reviewer's point about "restriction" being independent of CFU. In our use of the term, which we think is clear in the manuscript, "restriction" refers to changes in bacterial survival and replication. Measurement of CFU is the best, most sensitive,

and perhaps only way to measure “restriction”. We have revised the manuscript to be more clear that our use of the term “restriction” refers to a change (decrease) in CFU burdens.

The claim of a universal failure overlooks cell-specific roles. The data cannot rule out that the deleted genes have critical functions in specific myeloid subsets that are masked by their permissive role in other cell type hosting bacteria. Where are the bacteria at 14 dpi? Do these mutant mice have altered bacterial distribution? It is possible that these genes have function in specific subtypes of macrophages but not all the cells, therefore claiming a lack of function of entire innate immune control of Mtb may be overstating. For example, Myd88-/- affects the ability of Mtb infected alveolar macrophages to cross lung epithelial cells to recruit the monocytes, which affect monocytes mediated Mtb control as well as antigen trafficking and T cell activation.

These are all good questions, and we would have been much more interested in pursuing them in the mutant mice if we had seen differences in bacterial burdens. Many of the questions (location of bacteria in AMs, role of MyD88 on migration of bacteria to the interstitium, etc.) have been addressed in wild-type mice in publications from the Urdahl lab (PMID 30146391). We agree that some of these processes could be changed in the knockout mice, but we feel that these would then be “differences that do not make a difference” and so we did not prioritize experiments to characterize them. However, the reviewer make a good point that we cannot claim that there is absolutely no change in the innate response in the knockout mice. We have added a sentence to the manuscript (lines 340-343) that “These results do not rule out the possibility that innate immune cell activation, differentiation, survival or migration might be altered in the knockout mice; we can only conclude that even if such differences exist, they do not have a significant impact on the ability of the innate immune system to restrict bacterial burdens”.

At 14 dpi, the bacterial is likely predominantly located in alveolar macrophages (AM), as even with over 2000 CFU, AM stays as the predominant cell type where the bacterial replicates. Does AM play any role in controlling or Mtb replication in ultra-low dose? AM has limited ability to control Mtb in the absence of adaptive immune response. Is the unchanged CFU in mutant mice reflects the inertness of AM or independence of these pathways in AM to control early Mtb replication? How about monocytes derived macrophages?

At 14 dpi, the bacterial are generally located in alveolar macrophages both in the alveolar and interstitial lung space (PMID: 30146391). However, since we see no role of the innate immune system in restricting bacterial replication at this timepoint, we do not believe that AMs (or indeed any other innate cell type) are controlling the bacterial burdens. We do not believe that AMs are “inert” as they seem to have a potent ability to restrict other bacterial pathogens (e.g., Legionella, as documented in our manuscript, and by many other labs). We also do not believe that the activation state of the innate immune system explains its failure to restrict Mtb (this is a major point of our Lp priming experiments). Instead, as we discuss in the manuscript, we believe that the failure of the innate immune system to change bacterial burdens is due to the intrinsic resistance of

the bacteria to the intrinsic killing mechanisms of macrophages (AMs or otherwise). We feel this is already extensively discussed in the manuscript.

Is there any evidence that the tested pathways are activated in ultra-low dose model? Or does this mean ultra-low dose Mtb activates an innate immune pathway distinct from these known ones? Does the mutation in innate immune signaling lead to altered T cell activation dynamic or granuloma structure or Mtb dissemination to the other lobe and/or dLN? Innate immune activation consequence?

We agree that some of these tested pathways may not be sufficiently activated in the ultra-low dose model; in fact, IFNγ is unlikely to be produced at high levels this time point (though it is difficult to rule out lower levels of IFNγ production by innate cells). Due to the very low levels of bacteria in the lungs, conventional methods of cytokine production (e.g., by ELISA or even ICS) are not sensitive enough for us to determine if there is local production of these factors. However, several of these critical innate immune pathways (e.g., autophagy) are likely to be activated, given their documented role in the innate immune response to Mtb. Since the effects of ULD infection on adaptive immune responses to Mtb have been thoroughly investigated by Kevin Urdahl's lab (PMID: 33142108), the focus of our manuscript is on the innate response.

2. about pet-store mice: two methods were attempted to enhance innate immune responses to Mtb—pet shop mouse co-housing and by Lp co-infections. The link of pet shop mouse co-housing model to lung-specific innate immunity is not demonstrated. Pet-store mice co-housing model calibrates microbiota. However, the main effect of this model is to prime T cell response with an unclear tie with innate immune activation (this was also reflected in Figure 2). While showing co-housing with pet store mice were able to control systemic Listeria infection, there is no evidence that pet-store microbiota affected lung innate immunity. Indeed, Lp infection was not affected in the co-housed mice (with a small cage effect). What is the evidence that pet-store microbiota has successfully primed innate immunity in the lung? Listeria was a systemic infection that doesn't mainly target the lung. Do co-house mice have altered Listeria in the lung to compare Listeria to lung infections? Does this control rely on innate immunity?

These are all good questions and indeed the reviewer's main point—that pet shop cohousing may not induce innate immunity in the lung—is one that we made clearly in the initial manuscript ourselves. Indeed, that finding—which we feel is important for any future investigators interested in using the pet shop model—was precisely what motivated us to use Lp priming, which we demonstrate does induce potent innate immune antibacterial activity in the lung. We fully agree that the questions the reviewer is asking are excellent questions, but we feel that we already discussed and addressed these issues in our initial manuscript. However, we have made additional changes to further clarify the limitations of the pet shop model.

3. about Lp priming: This part distracts from the main finding rather than providing conceptual insight. Does pet-store microbiota prime innate immunity through the same mechanism as Lp?

We are somewhat confused by this comment and question. We feel that the Lp priming experiments are central to the manuscript. Indeed, the reviewer notes above that pet shop priming is only able to poorly protect against lung Lp infection, suggesting that it does not enhance innate immunity. By contrast, we see very robust protection against Lp lung infection that is stimulated by prior Lp priming (see Figure 4). Thus, we feel that we have documented that pet shop priming and Lp priming have very different effects; and indeed, the lack of a strong effect of pet shop priming and a strong effect of Lp priming was what motivated us to pursue Lp priming as an additional method to stimulate innate immunity in the lung. We feel the Lp priming experiments are thus central to the paper, not a distraction.

Lp (even deltaFlaA) triggers neutrophil and monocyte infiltration and AM reduction. Why would the author think that these changes would even affect Mtb infection?

We hypothesized that Lp priming would enhance control of Mtb control because we observed that Lp priming does activate the innate immune system and (as evidence that this activation is functional) highly enhances control of Lp infection (see Figure 4). The interesting result we obtained, though, is that this enhancement of innate immunity did not significantly impact Mtb burdens in the lung, leading to our conclusion that Mtb exhibits resistance even to an enhanced innate immune response in the lung.

The results are under-analyzed.

Naturally, we disagree with this statement; indeed, we performed detailed characterization of the response, including by scRNA-seq (and, as new data to the manuscript, by histology and cytokine responses). We are not sure what the reviewer is asking for.

This is a low-dose infection. In this model, monocytes have been shown to restrict Mtb replication. However, in this study the Mtb CFU was unaltered with monocytes infiltration, why did the monocytes failed to do so? Are the monocytes recruited by Lp different from those during Mtb infection? Do the altered innate cell population lead to altered Mtb distribution as a result of innate control? Lp priming altered gene expression and cell types in the lung after Mtb infection. Meaning that innate immune response is likely changed.

We agree with the reviewer that monocyte-derived macrophages have previously been shown to restrict Mtb replication; however, this has been shown to be the case only after the onset of adaptive immunity. It was not clear whether the innate system could restrict Mtb prior to the onset of adaptive immunity, which is the question we sought to address. The interesting result we provide is that monocytes (or indeed the entire innate response) is insufficient to control Mtb, even after Lp priming (whereas primed innate cells exhibit markedly enhanced control a subsequent Lp challenge). We expend considerable effort to address what is happening after Lp priming, including a detailed analysis of the responding cells by scRNA-seq. This analysis shows that indeed the monocytes are activated after Lp priming; so we can rule out the trivial explanation that the monocytes fail to become activated.

What does this cause other than NOT reducing CFU?

Our scRNA-seq data document many changes to the myeloid cell compartment after Lp priming.

Does it affect T cell priming and control of Mtb?

We feel that we were very clear about the scope of the manuscript being the question of whether the *innate* immune system can restrict Mtb replication. This is not a question that has been extensively investigated to date, whereas many other papers have addressed the important role of the adaptive immune response in Mtb control. We feel that an investigation of the adaptive immune response after Lp priming may be an interesting question, but it is not the question we sought to address in our manuscript. It is also a very challenging question to address since both innate and adaptive immune responses are present during the adaptive phase of the response and are very challenging to disentangle.

Why does Lp affect Mtb dissemination to the spleen?

This is an interesting question that we do not know the answer to. Since we don't see a strong decrease in the number of CFU in the lung after Lp priming, we infer that the decreased levels of Mtb in the spleen must be due to decreased dissemination, or perhaps decreased replication or survival in the spleen. Our scRNA-seq data indicate that unprimed mice express a stronger interferon response upon Mtb infection. While it is unclear from the expressed ISGs if this interferon response is due to either IFN-γ or IFN-β signaling, we have hypothesized that perhaps the increased inflammatory response in the unprimed mice has resulted in increased dissemination. Although this question is certainly interesting, to address it fully would require substantial experimentation that would extend well beyond the intended focus of our study which was to evaluate the role of innate immunity in restricting Mtb burdens in the lung.

Reviewer #3: My main concerns are the following
1- the ultra-low dose model used is a tricky one and may induce strong biases in interpretation. Indeed, in the different experiments used there is a large - and highly variable- ratio of animals that are not infected due to low dose exposure. Yet these animals have been exposed, so it is highly possible that they have eliminated the bacteria which would be via innate immune mechanisms. I understand that the authors used a variety of KO animals to address this concern, making the hypothesis that in these Ko mice, innate mechanisms would be impaired, and this would make a difference. Again, in that case an important ratio of KO mice showed no sign of infection despite exposure, which again could be interpreted as a sign of early innate control. It is highly possible that redundant innate mechanisms are at play to control early Mtb multiplication at these early stages and using single KO animals does not really help to address this question.

We fully agree with the reviewer about the technical trickiness of the ultra-low dose model. We believe we have done everything correctly and that our interpretation of these experiments is appropriate. Although there is indeed some variation in the fraction of animals infected in different experiments, we were actually impressed that we were always able to infect cohorts of mice such that some mice were always infected, but the fraction infected was always less than 70% (the Poisson distribution threshold at which we can infer that the mice received 1-3 bacteria (see prior calculations from the Urdahl Lab (PMID: 33142108) and Frothingham Lab (PMID: 22197183)). Given that less than a 2-fold difference in inoculum could have either resulted in almost no infection, or non-ultra-low dose infections (>1-3 bacteria/mouse), we were proud that we were able to optimize the conditions such that we were able to consistently achieve ultra-low dose infections.

The reviewer is correct that it is formally possible that some mice are infected and immediately clear the infection and that we would score such mice as "uninfected" rather than resistant. As the reviewer appreciates, this is the reason why it was important for us to control our experiments by comparing different genotypes of mice infected in parallel during the same aerosolization procedure. Our key finding that wild-type and knockout mice are not infected at significantly different ratios implies that the specific immune pathway present in the wild-type mice (but absent in the knockouts) does not provide an essential innate immune mechanism by which to clear Mtb at early time points. Although redundancy is always a possibility when using knockout mice (this is true for conventional infections as well), we chose immune factors that are known to provide non-redundant control of Mtb during the adaptive phase of the infection and that are indeed the main factors known to mediate resistance to Mtb (e.g. TNF, IFNγ, MyD88, Atg5, etc). Thus, we can conclude that these known, critical factors surprisingly play no detectable role during the innate phase of the response, even in a highly sensitized ultra-low dose infection. We think this is an important conclusion even if it remains possible there are (unknown) factors we didn't test that do mediate some bacterial control. Nevertheless, to address the reviewers concern, we have added language to the text to acknowledge the possibility that redundant mechanisms may

have prevented us from identifying innate immune pathways that can control Mtb replication (lines 338-346 of the revised manuscript).

2- i am not entirely convinced that exposure of mice to mouse pathogens by co-housing with pet shop mice or by Lp infection can be accepted as a good model to only activate the innate immune system. In fact, such infections make the animals sick -as actually shown by weight loss (supplemental figure 1). So could such treatment induce immunosuppression -even temporary- rather than a simple "activation of the innate immune system". This would render interpretation of the data much trickier.

We fully agree with the reviewer that pet shop co-housing is complex and may have multiple immunostimulatory and immunosuppressive effects, and that these effects could cancel out. Indeed, pet shop co-housing does not only activate the innate immune system, as we see enrichment of effector memory T cells in the pet shop co-housed mice. Indeed, we discussed this and other limitations of the pet shop infection model in our original manuscript (lines 184-190 of the revised manuscript). We do confirm that the pet shop co-housed mice are resistant to *Listeria* infection (as previously shown by others), which, at a minimum, rules out the possibility that they are broadly immunosuppressed. However, as discussed above in response to reviewer 2 (point 2), we can find no evidence that pet shop priming promotes resistance to bacterial infections in the lung. Indeed, this is an important finding for future investigators planning to use the pet shop model. The lack of a pet shop-primed antibacterial response in the lung is precisely what motivated us to switch to Lp priming, where we do see primes a very robust anti-bacterial response (Figure 4) that nevertheless fails to impact Mtb control.

3- As a more general comment, I was under the impression that the authors did not really consider the real pathophysiology of Mtb infection that is much more than CFUs counts in the lung. It is not surprising that Mtb is not cleared from the lung tissue as it is this way of living. There is, to my knowledge, no real consensus that any Mtb infection may be sterilizing, even in humans, except very long after exposure which is not the case here in the short-term models used. I would have liked to see lesions in lungs for example. Are granuloma formed the same way in the different settings?

The reviewer is correct that our initial manuscript focused almost entirely on restriction of bacterial burdens and did not consider the contribution of innate immunity to pathophysiology. This is a comment made by reviewers 1 and 2 as well and we thank the reviewers for pushing us to better highlight this question. Although there is little pathology to observe at the day 14 timepoint, we have added several additional analyses (histology, cytokines) and discussion of this point to the manuscript, as already discussed extensively above.

To our knowledge, there is little evidence that the innate immune system can clear Mtb in humans and we have added to the discussion on this point.

Part III – Minor Issues: Editorial and Data Presentation Modifications
Please use this section for editorial suggestions as well as relatively minor modifications of existing data that would enhance clarity.

Reviewer #1: Minor Comments:
1. The limitation inherent to the analysis of bystander cells only in the scRNA-seq dataset should be more clearly emphasized in the Results and Discussion sections.
2. Additional clarification regarding upstream pathways contributing to the interferon-stimulated gene signature would improve interpretability.
3. Several statements in the Discussion equate inflammation with antimicrobial efficacy and would benefit from more precise wording.
4. The possibility of very early innate effects (hours to days) that are not captured by day-14 CFU analysis should be acknowledged.
5. When discussing type I interferon responses, a stronger linkage to clinical data associating elevated IFN signatures with poor TB outcomes would improve translational relevance.
6. Figures presenting flow cytometry and scRNA-seq data should emphasize variability and overlap between groups to avoid over-interpretation of modest differences.
7. Data availability and accessibility: The manuscript reports the generation of OMIC-scale datasets, including scRNA-seq data, but does not provide accession numbers or a clear statement on public data deposition. In accordance with PLOS Pathogens data availability policies, the authors should specify the repository and corresponding accession number(s), or indicate that the data will be made publicly available upon publication.

We have added text or clarified language in the Results and Discussion as suggested by the reviewer. We have also updated our text to include information on data accessibility and availability.

Reviewer #2: Minor suggestions:

139, missing a comma after CA
160, 167, CFUs or CFU?
260, ISG, stimulated instead of “stimulate”
269, data revealed instead of “reveals”
528, fig1A, missing the median bar for C57BL/6 group.

The methodology does not contain sufficient details. For example, Listeria infection model is not found in the method.

Some description is not accurate. For example, line 174 “co-housing induced small changes to the composition of immune cells in the lung by day 14 post-infection”, changes were observed before infection.

We have updated the text to correct these errors.

Reviewer #3: As a complement to the last point above, the authors make an important finding that Lp exposure prior to Mtb infection limits spreading to the spleen: this is not commented that much, and I actually believe that this finding is more relevant to the general role of innate immunity to the development of Mtb pathophysiology.

We have expanded our discussion of the effects of Lp priming on Mtb CFU burden in the spleen (lines 360-368 of the revised manuscript).
