# Supplementary material for: Diverse infection models demonstrate robust resistance of Mycobacterium tuberculosis to innate immunity
Source: PLoS Pathog. 2026 Oct 21;22(10):e1014713. doi: 10.1371/journal.ppat.1014713 (PMC13705603; doi:10.1371/journal.ppat.1014713)
Supplement: Supplementary file 2 [file ppat.1014713.s011.pdf]

PPATHOGENS-D-25-03003R1
Diverse infection models demonstrate robust resistance of Mycobacterium tuberculosis to innate immunity

We thank the Editor and Reviewers for this thoughtful round of reviews. We are pleased that the reviewers appreciated the conceptual advance of our work and that there were no further experimental requests and that the suggested revisions include modifications to the text. We have endeavored to explain our modifications below.

As requested, our revised manuscript submission includes:

1. This “Response to Reviewers” that addresses each point raised by the reviewers (see below).

2. A marked-up copy of our manuscript labeled 'Revised Manuscript with Track Changes' that highlights changes made to the original version.

3. An unmarked version of our revised paper without tracked changes labeled 'Manuscript'.

Reviewers' Comments:

Part I - Summary

Reviewer #1: The authors have addressed all my major concerns conceptually and experimentally.

Reviewer #2: The conceptual advance of this study is that Mtb remains remarkably resistant to innate immune-mediated clearance in vivo, even under conditions in which innate immunity is broadly activated (Mtb infection itself, microbiota-educated hosts, or prior Legionella infection). This has important implications for how the field interprets macrophage antimicrobial assays. Numerous studies report altered bacterial growth in cultured macrophages following manipulation of specific innate pathways, yet it often remains unclear whether these effects translate into physiologically meaningful protection in vivo. I encourage the authors to emphasize this distinction more explicitly in the Abstract and Discussion. Specifically, the manuscript would benefit from a thorough discussion that cell-intrinsic restriction of Mtb growth in isolated macrophages does not necessarily predict host-level control of infection in the lung.

Response: We have endeavored to better emphasize the point suggested by the reviewer. Specifically, see changes we have made on lines 18-22 of the abstract and lines 427-431 in the Discussion.

A related conceptual question also deserves discussion. If innate immunity has limited capacity to directly reduce Mtb bacterial burden, how do we interpret data reporting Mtb may evade or impair innate immunity, what's the benefit of doing so for the bacteria. And how should we interpret innate pathways that clearly influence disease susceptibility? Discussing this distinction would help place the present findings into the broader context of tuberculosis immunology.

Response: The reviewer’s comments suggest that perhaps we need to be clearer on two key points: (1) we believe that the reason innate immunity has a limited capacity to reduce Mtb burdens is not because innate immunity is generally ineffective. Indeed, as we document in the manuscript, innate immune mechanisms in the lung are highly effective in eliminating other

bacteria, such as *Legionella*. Instead, we believe the reason innate immunity has limited capacity to reduce Mtb specifically is because Mtb has evolved intrinsic resistance to its effector mechanisms. There is clear benefit to the bacteria to evolving such resistance—indeed we think it is one of the key reasons hosts fail to sterilize Mtb infections. (2) We do not believe that innate immune pathways play no role whatsoever in resistance to Mtb. Our point is only that innate immunity alone is insufficient to control Mtb (prior to the emergence of adaptive immunity at ~2 weeks post infection). Once adaptive immunity is engaged, it is clear that many of the innate pathways we discuss (especially IFNγ-induced antibacterial killing by macrophages) are key to Mtb control. Thus, innate immunity clearly influences disease susceptibility; but at the same time, it is insufficient, by itself, to impact bacterial burdens. We have tried to more clearly make these points in lines 319-326.

Reviewer #3: The strength of this study is an original question : i.e. "does M tuberculosis does resist to innate immunity"? Yet, the weakness of the study remains in the models used that are very complicated and do vary along the study (for example the beginning is carried out with 1-3 ultra low dose infection and then the authors shift to 50 CFUs without further explanation). The readouts remain limited and there are still some potential pitfalls with i) the ULD model (and variable outcome of infection which could lead to misinterpretation) and ii) and interpretation of some immunological data. For example, the fact that Lp primed mice do control secondary infection better (see Figure 4A) could well be explained by Lp-specific onset of adaptive immunity making the sentence " This implies that priming with Lp strongly activates an anti-bacterial innate immune response" (line 208-209) an overstatement.

Response: The reviewer makes several points:
(a) **“the models are complex”**—yes, we agree. We believe we have done a good job of discussing their complexities and limitations throughout the manuscript.
(b) **“the models used...vary along the study” and we shift from using an ULD infection model to standard infection doses without explanation.** Yes, we do use a variety of infection models in our study. However, we believe using a variety of models to reinforce our conclusions is a strength not a weakness of the manuscript. We now briefly explain on lines 175-177 that we could not use the ULD in combination with pet shop co-housing because in the ULD model, approximately half of the mice are not infected, and thus the number of infected mice co-housed with each pet shop mouse would fall below the numbers of mice necessary for sufficiently statistically powered comparisons, bearing in mind that a substantial fraction of co-housed mice die after exposure to pet shop mice, and in addition, we could not combine cages of pet shop co-housed mice because each pet shop mouse tended to give unique results.
(c) **“readouts remain limited”**—we performed many different kinds of analyses in the paper including CFU, histology, cytokine analyses, flow cytometry, and scRNA-seq, etc. We aren’t sure what readouts the reviewer would have preferred to see or why such readouts were not requested in the previous round of review. In addition, we aren’t sure what conclusions any additional readouts would have allowed us to make that we don’t already make. The revised version of the paper did add a considerable number of requested readouts to the analysis, and these did not vastly change the conclusions we were able to make.
(d) **“the fact that Lp primed mice do control secondary infection better (see Figure 4A) could well be explained by Lp-specific onset of adaptive immunity”**—the control of a secondary Lp infection that is elicited by a primary Lp pre-infection is very rapid (it occurs between days 2-4 after primary Lp infection). We think it is highly unlikely this is due to adaptive immunity, but acknowledge that it is a formal possibility. We now discuss this point in the revised manuscript on line 226-227.

Part II – Major Issues: Key Experiments Required for Acceptance
Please use this section to detail the key new experiments or modifications of existing experiments that should be absolutely required to validate study conclusions.

Generally, there should be no more than 3 such required experiments or major modifications for a "Major Revision" recommendation. If more than 3 experiments are necessary to validate the study conclusions, then you are encouraged to recommend "Reject".

Reviewer #1: The authors have addressed all my major issues, including key experiments, and the manuscript has improved dramatically in data interpretation, quality of results, and deeper discussion that accepts limitations and open possibilities. Therefore, this reviewer deems this manuscript fit for publication.
Reviewer #2: (No Response)
Reviewer #3: The revised version of the manuscript addresses most of my concerns even though to my opinion some data remain over interpreted (see above)

Response: we are glad that the reviewers have not requested additional experimentation.

Part III – Minor Issues: Editorial and Data Presentation Modifications
Please use this section for editorial suggestions as well as relatively minor modifications of existing data that would enhance clarity.

Reviewer #1: All minor issues have been addressed in the new version of the manuscript.
Reviewer #2: (No Response)
Reviewer #3: IN figure SFig4 it would be much more confortable to read the name of each mediator on top of the graph rather than looking for this information in the figure caption.

Response: We have made the change suggested by the review and agree this is a nice improvement. Thank you!
