# Supplementary material for: Diverse infection models demonstrate robust resistance of Mycobacterium tuberculosis to innate immunity
Source: PLoS Pathog. 2026 Oct 21;22(10):e1014713. doi: 10.1371/journal.ppat.1014713 (PMC13705603; doi:10.1371/journal.ppat.1014713)
Supplement: Peer review history

# Peer review: Decision Letter 0

**DOI:** 10.1371/journal.ppat.1014713.r001

**Authors:** Thomas Guillard, Pierre Santucci

19 Jan 2026

PPATHOGENS-D-25-03003

Diverse infection models demonstrate robust resistance of Mycobacterium tuberculosis to innate immunity

PLOS Pathogens

Dear Dr. Vance,

Thank you for submitting your manuscript to PLOS Pathogens. After careful consideration, we feel that it has merit but does not fully meet PLOS Pathogens's publication criteria as it currently stands. Therefore, we invite you to submit a revised version of the manuscript that addresses the points raised during the review process.

Please submit your revised manuscript by Mar 20 2026 11:59PM. If you will need more time than this to complete your revisions, please reply to this message or contact the journal office at plospathogens@plos.org. When you're ready to submit your revision, log on to https://www.editorialmanager.com/ppathogens/ and select the 'Submissions Needing Revision' folder to locate your manuscript file.

Please include the following items when submitting your revised manuscript:

* A letter that responds to each point raised by the editor and reviewer(s). You should upload this letter as a separate file labeled 'Response to Reviewers'. This file does not need to include responses to any formatting updates and technical items listed in the 'Journal Requirements' section below.

* A marked-up copy of your manuscript that highlights changes made to the original version. You should upload this as a separate file labeled 'Revised Manuscript with Track Changes'.

* An unmarked version of your revised paper without tracked changes. You should upload this as a separate file labeled 'Manuscript'.

If you would like to make changes to your financial disclosure, competing interests statement, or data availability statement, please make these updates within the submission form at the time of resubmission. Guidelines for resubmitting your figure files are available below the reviewer comments at the end of this letter.

We look forward to receiving your revised manuscript.

Kind regards,

Pierre Santucci

Academic Editor

PLOS Pathogens

Thomas Guillard

Section Editor

PLOS Pathogens Sumita Bhaduri-McIntosh

Editor-in-Chief

PLOS Pathogens

orcid.org/0000-0003-2946-9497

Michael Malim

Editor-in-Chief

PLOS Pathogens

orcid.org/0000-0002-7699-2064

**Additional Editor Comments:**

Dear Dr. Vance,

Thank you for submitting your manuscript to PLOS Pathogens. After careful evaluation of your manuscript by members of the editorial board and external experts, we would like to invite you to submit a revised version of your work for further consideration. While the reviewers find the topic of your work to be timely, well-executed and of great interest for the readership of PLOS Pathogens, they have raised several concerns and limitations that must be addressed in a revised version before the paper can be accepted for publication. Some of these concerns will require experimental validation, as you will see the three reviewers have acknowledged that complementary approaches to CFU counting is required to back up your interpretation and strengthen the conclusions of your manuscript. Of note, some of the experts raised concerns about data accessibility. Please make sure to follow PLOS Pathogens guidelines regarding data accessiblity and reproducibility in your revised version by providing all the necessary information and access to the appropriate datasets.

Thank you again for considering PLOS Pathogens for your work. We look forward to receiving your revised manuscript.

Yours sincerely,

Pierre Santucci

Academic Editor

PLOS Pathogens

**Journal Requirements:**

If the reviewer comments include a recommendation to cite specific previously published works, please review and evaluate these publications to determine whether they are relevant and should be cited. There is no requirement to cite these works unless the editor has indicated otherwise.

1) Please ensure that the CRediT author contributions listed for every co-author are completed accurately and in full.

At this stage, the following Authors/Authors require contributions: Marian R Fairgrieve, Ella C Brydon, Roberto A Chavez, Dmitri I Kotov, and Russell Vance. Please ensure that the full contributions of each author are acknowledged in the "Add/Edit/Remove Authors" section of our submission form.

The list of CRediT author contributions may be found here: https://journals.plos.org/plospathogens/s/authorship#loc-author-contributions

2) We ask that a manuscript source file is provided at Revision. Please upload your manuscript file as a .doc, .docx, .rtf or .tex. If you are providing a .tex file, please upload it under the item type u2018LaTeX Source Fileu2019 and leave your .pdf version as the item type u2018Manuscriptu2019.

3) Please upload all main figures as separate Figure files in .tif or .eps format. For more information about how to convert and format your figure files please see our guidelines:

https://journals.plos.org/plospathogens/s/figures

4) We notice that your supplementary Figures, and Tables are included in the manuscript file. Please remove them and upload them with the file type 'Supporting Information'. Please ensure that each Supporting Information file has a legend listed in the manuscript after the references list.

5) Some material included in your submission may be copyrighted. According to PLOSu2019s copyright policy, authors who use figures or other material (e.g., graphics, clipart, maps) from another author or copyright holder must demonstrate or obtain permission to publish this material under the Creative Commons Attribution 4.0 International (CC BY 4.0) License used by PLOS journals. Please closely review the details of PLOSu2019s copyright requirements here: PLOS Licenses and Copyright. If you need to request permissions from a copyright holder, you may use PLOS's Copyright Content Permission form.

Please respond directly to this email and provide any known details concerning your material's license terms and permissions required for reuse, even if you have not yet obtained copyright permissions or are unsure of your material's copyright compatibility. Once you have responded and addressed all other outstanding technical requirements, you may resubmit your manuscript within Editorial Manager.

Potential Copyright Issues:

i) Figures 2A, 4A, 4C, 4G, and 5A. Please confirm whether you drew the images / clip-art within the figure panels by hand. If you did not draw the images, please provide (a) a link to the source of the images or icons and their license / terms of use; or (b) written permission from the copyright holder to publish the images or icons under our CC BY 4.0 license. Alternatively, you may replace the images with open source alternatives. See these open source resources you may use to replace images / clip-art:

- https://commons.wikimedia.org

- https://openclipart.org/.

6) Please amend your detailed Financial Disclosure statement. This is published with the article. It must therefore be completed in full sentences and contain the exact wording you wish to be published.

1) State the initials, alongside each funding source, of each author to receive each grant. For example: "This work was supported by the National Institutes of Health (####### to AM; ###### to CJ) and the National Science Foundation (###### to AM)."

2) State what role the funders took in the study. If the funders had no role in your study, please state: "The funders had no role in study design, data collection and analysis, decision to publish, or preparation of the manuscript."

3) If any authors received a salary from any of your funders, please state which authors and which funders..

If you did not receive any funding for this study, please simply state: u201cThe authors received no specific funding for this work.u201d

7)  Please ensure that the funders and grant numbers match between the Financial Disclosure field and the Funding Information tab in your submission form. Note that the funders must be provided in the same order in both places as well.

8) Please send a completed 'Competing Interests' statement, including any COIs declared by your co-authors. If you have no competing interests to declare, please state "The authors have declared that no competing interests exist". Otherwise please declare all competing interests beginning with the statement "I have read the journal's policy and the authors of this manuscript have the following competing interests"

**Reviewers' Comments:**

Reviewer's Responses to Questions

**Part I - Summary**

Please use this section to discuss strengths/weaknesses of study, novelty/significance, general execution and scholarship.

Reviewer #1: Summary:

In this manuscript, Fairgrieve et al. examine whether innate immune mechanisms can restrict Mycobacterium tuberculosis (Mtb) during early infection. Using ultra-low dose aerosol infection, environmental immune priming, and lung-restricted inflammation induced by Legionella pneumophila, the authors conclude that Mtb is intrinsically resistant to innate immune clearance. The study is technically sound and addresses an important, long-standing question in tuberculosis immunology. However, the conclusions rely almost entirely on CFU-based measurements and, in doing so, underinterpret several observations that point to altered containment and disease tolerance mechanisms.

Major Comment:

Innate immune priming, dissemination, and disease tolerance require tissue-level analysis:

The central conclusion that innate immunity has a limited capacity to control Mtb is primarily based on pulmonary CFU measurements. While this interpretation is technically correct, it is overly restrictive. It does not fully account for an important result in the study: priming innate immunity with Legionella pneumophila significantly reduces extrapulmonary dissemination of Mtb to the spleen, despite no detectable change in lung CFUs. Reduced dissemination is a biologically meaningful phenotype and should not be considered secondary. Rather, it suggests that innate immune priming alters mechanisms of bacterial containment instead of directly enhancing bacterial killing.

This interpretation is further supported by the CD64-positive enriched scRNA-seq analysis, which identifies an increased representation of CD86-positive myeloid cells following Legionella priming. These cells are not classically associated with direct antimicrobial activity. Instead, they clearly show scRNA-seq gene signatures linked to regulation of immune responses, resolution of inflammation, wound healing, regulation of vascular development, lipid and cholesterol handling, including APOE-associated pathways, and metabolic homeostasis, such as responses to insulin signaling. Taken together, these transcriptional features are consistent with a shift toward disease tolerance, in which host tissues limit damage and spatially constrain infection, rather than with enhanced bactericidal activity (references 1-2).

Despite these indications, the manuscript does not experimentally address whether innate immune priming alters lung tissue architecture, inflammatory damage, immune organization, or routes of dissemination. As a result, the conclusion that innate immunity plays only a limited role in controlling Mtb risks being overly CFU-centric and conceptually incomplete.

Bibliography:

1. Ayres, J.S., Schneider, D.S. Tolerance of infections. (2012) Annual Review of Immunology. 30:271-94. DOI: 10.1146/annurev-immunol-020711-075030.

2. Medzhitov R, Schneider DS, Soares MP. Disease tolerance as a defense strategy. Science. 2012 Feb 24;335(6071):936-41. doi: 10.1126/science.1214935. PMID: 22363001; PMCID: PMC3564547.

Reviewer #2: In this manuscript, Farigrieve et al. investigates a central and intriguing question in TB immunology: whether pre-existing or infection-induced innate immune activation can control Mycobacterium tuberculosis (Mtb). Prior literature has been showing that innate immunity was less efficient to control Mtb infection, but the findings have not been properly summarized. The authors here provided a summary of the failed control of Mtb replication by innate immunity, by reporting that while innate immune activation controls Listeria or Lp, it fails to restrict Mtb growth. The question is significant, but the study’s ambitious conclusion—that the innate immune system cannot restrict Mtb—needs future support. The authors failed to go much beyond CFU measure or to provide mechanistic insights. This lack of deeper investigation limits the novelty and impact. Overall, the conclusion needs more evidence to support. It would be interesting to investigate what is the “innate immunity” activated by ultra-low dose or regular does Mtb? I am also wondering: why does this form of innate immunity fail to restrict Mtb? Does another type of innate immunity exist to restrict Mtb replication?

Reviewer #3: The study presented here using different mouse models and set up wishes to address the role of the innate immune defense in the lung against Mycobacterium tuberculosis (Mtb) infection. The authors wish to challenge the concept that local inflammation raised by Mtb infection itself or by co-housing with pet-shop mice or prior controlled infection with another lung pathogen (here Legionella pneumophila/Lp) that activates innate cells -before the onset of adaptive immunity- should be able to eradicate or clear Mtb. The main findings and claims are that primed innate cells in vivo in the lung do no control Mtb multiplication as no difference in CFUs are measured between SPF wild type mice (as commonly used by many other laboratories) and all other mice used (knock-out , co-housed, Lp infected etc...).

There is a consensus that some exposed individuals might be able to resist Mtb infection and deciphering the mechanisms involved is a very important issue that may help to better understand Mtb infection pathophysiology and epidemiology. In that respect the study is timely and important. However, I am not convinced that the methods and data presented really shed new light on the role of innate immunity in Mtb control.

**********

**Part II – Major Issues: Key Experiments Required for Acceptance**

Please use this section to detail the key new experiments or modifications of existing experiments that should be absolutely required to validate study conclusions.

Generally, there should be no more than 3 such required experiments or major modifications for a "Major Revision" recommendation. If more than 3 experiments are necessary to validate the study conclusions, then you are encouraged to recommend "Reject".

Reviewer #1: To support a more comprehensive interpretation, the authors should include tissue-based and inflammatory analyses that are well within the scope of the current study. These could include:

1) Histological analysis:

-H&E staining to assess lung inflammation, tissue damage, and lesion organization, including granuloma aggregates.

- Spatial localization of Mtb within lung compartments using acid-fast staining or fluorescence-based approaches, where applicable.

- Immunohistochemistry or immunofluorescence to evaluate myeloid and lymphoid organization associated with containment.

2) Measurement of inflammatory mediators in lung homogenates or bronchoalveolar lavage fluid (if available), such as IL-1beta, TNF, IL-6, CXCL1, CXCL2, CCL2, and IL-10.

3) Targeted qPCR analyses from Trizol-preserved lung tissue to assess inflammatory mediators (Il1b, Tnf, Il6), type I interferon and interferon-stimulated gene programs (Ifnb1, Isg15, Mx1), macrophage activation or tolerance markers (Nos2, Arg1, Apoe), and markers of lung injury or dysfunction (Hmgb1, Mmp9, Mmp12, Sftpc).

These experiments would allow the authors to directly test whether innate immune priming reshapes infection topology and host tolerance, providing a mechanistic explanation for reduced dissemination despite unchanged lung bacterial burden.

Reviewer #2: 1. The ultra-low dose model: The conclusion on that key innate immunity genes known to control Mtb control do not exert substantial effects during the innate phase of the infection, even at highly limited infectious doses is important. The main observation is that the CFU of Mtb was not changed after ultra-low dose infection in all immune deficient mice models. Mice with deletion in Ifngr1, TNFR, Sp140, Myd88/Trif, or harboring Atg5 myeloid deficiency all showed comparable bacterial load at 14 dpi after ultra-low dose Mtb challenge, similar as observed in standard low-dose infection (as cited by the authors and PMID: 37319285). Since the unrestricted control of bacilli growth in innate cells has been reported, the data presented serve to confirm, rather than advance, established knowledge with a newer model. In the standard low-dose model, the failure to control Mtb CFU in these immune deficient mice was observed later at the onset of T cell response. How about in ultra-low dose model?

Innate immunity has many facets. Directly restriction bacterial replication is only one of it. Therefore, unchanged CFU may not reflect a complete failure of innate immunity. The innate immunity perhaps is slow in restricting Mtb. 14 dpi CFU provided a snapshot. Not controlling at this timepoint doesn’t mean it fails to control infection. It is possible that the “Mtb restriction” is happening in a way undetectable by log changes of CFU. The claim of a universal failure overlooks cell-specific roles. The data cannot rule out that the deleted genes have critical functions in specific myeloid subsets that are masked by their permissive role in other cell type hosting bacteria. Where are the bacteria at 14 dpi? Do these mutant mice have altered bacterial distribution? It is possible that these genes have function in specific subtypes of macrophages but not all the cells, therefore claiming a lack of function of entire innate immune control of Mtb may be overstating. For example, Myd88-/- affects the ability of Mtb infected alveolar macrophages to cross lung epithelial cells to recruit the monocytes, which affect monocytes mediated Mtb control as well as antigen trafficking and T cell activation.

At 14 dpi, the bacterial is likely predominantly located in alveolar macrophages (AM), as even with over 2000 CFU, AM stays as the predominant cell type where the bacterial replicates. Does AM play any role in controlling or Mtb replication in ultra-low dose? AM has limited ability to control Mtb in the absence of adaptive immune response. Is the unchanged CFU in mutant mice reflects the innertness of AM or independence of these pathways in AM to control early Mtb replication? How about monocytes derived macrophages?

Is there any evidence that the tested pathways are activated in ultra-low dose model? Or does this mean ultra-low dose Mtb activates an innate immune pathway distinct from these known ones? Does the mutation in innate immune signaling lead to altered T cell activation dynamic or granuloma structure or Mtb dissemination to the other lobe and/or dLN? Innate immune activation consequence?

2. about pet-store mice: two methods were attempted to enhance innate immune responses to Mtb—pet shop mouse co-housing and by Lp co-infections. The link of pet shop mouse co-housing model to lung-specific innate immunity is not demonstrated. Pet-store mice co-housing model calibrates microbiota. However, the main effect of this model is to prime T cell response with an unclear tie with innate immune activation (this was also reflected in Figure 2). While showing co-housing with pet store mice were able to control systemic Listeria infection, there is no evidence that pet-store microbiota affected lung innate immunity. Indeed, Lp infection was not affected in the co-housed mice (with a small cage effect). What is the evidence that pet-store microbiota has successfully primed innate immunity in the lung? Listeria was a systemic infection that doesn’t mainly target the lung. Do co-house mice have altered Listeria in the lung to compare Listeria to lung infections? Does this control rely on innate immunity?

3. about Lp priming: This part distracts from the main finding rather than providing conceptual insight. Does pet-store microbiota prime innate immunity through the same mechanism as Lp? Lp (even deltaFlaA) triggers neutrophil and monocyte infiltration and AM reduction. Why would the author think that these changes would even affect Mtb infection? The results are under-analyzed. This is a low-dose infection. In this model, monocytes have been shown to restrict Mtb replication. However, in this study the Mtb CFU was unaltered with monocytes infiltration, why did the monocytes failed to do so? Are the monocytes recruited by Lp different from those during Mtb infection? Do the altered innate cell population lead to altered Mtb distribution as a result of innate control? Lp priming altered gene expression and cell types in the lung after Mtb infection. Meaning that innate immune response is likely changed. What does this cause other than NOT reducing CFU? Does it affect T cell priming and control of Mtb? Why does Lp affect Mtb dissemination to the spleen?

Reviewer #3: My main concerns are the following

1- the ultra low dose model used is a tricky one and may induce strong biases in interpretation. Indeed, in the different experiments used there is a large - and highly variable- ratio of animals that are not infected due to low dose exposure. Yet these animals have been exposed , so it is highly possible that they have eliminated the bacteria which would be via innate immune mechanisms. I understand that the authors used a variety of KO animals to address this concern, making the hypothesis that in these Ko mice, innate mechanisms would be impaired and this would make a difference. Again in that case an important ratio of KO mice showed no sign of infection despite exposure, which again could be interpreted as a sign of early innate control. It is highly possible that redundant innate mechanisms are at play to control early Mtb multiplication at these early stages and using single KO animals does not really help to address this question.

2- i am not entirely convinced that exposure of mice to mouse pathogens by co-housing with pet shop mice or by Lp infection can be accepted as a good model to only activate the innate immune system. In fact such infections make the animals sick -as actually shown by weight loss (supplemental figure 1). So could such treatment induce immunosuppression -even temporary- rather than a simple "activation of the innate immune system" . This would render interpretation of the data much trickier.

3- As a more general comment, I was under the impression that the authors did not really consider the real pathophysiology of Mtb infection that is much more than CFUs counts in the lung. It is not surprising that Mtb is not cleared from the lung tissue as it is this way of living. There is, to my knowledge, no real consensus that any Mtb infection may be sterilizing, even in humans, except very long after exposure which is not the case here in the short-term models used. I would have liked to see lesions in lungs for example. Are granuloma formed the same way in the different settings?

**********

**Part III – Minor Issues: Editorial and Data Presentation Modifications**

Please use this section for editorial suggestions as well as relatively minor modifications of existing data that would enhance clarity.

Reviewer #1: Minor Comments:

1. The limitation inherent to the analysis of bystander cells only in the scRNA-seq dataset should be more clearly emphasized in the Results and Discussion sections.

2. Additional clarification regarding upstream pathways contributing to the interferon-stimulated gene signature would improve interpretability.

3. Several statements in the Discussion equate inflammation with antimicrobial efficacy and would benefit from more precise wording.

4. The possibility of very early innate effects (hours to days) that are not captured by day-14 CFU analysis should be acknowledged.

5. When discussing type I interferon responses, a stronger linkage to clinical data associating elevated IFN signatures with poor TB outcomes would improve translational relevance.

6. Figures presenting flow cytometry and scRNA-seq data should emphasize variability and overlap between groups to avoid over-interpretation of modest differences.

7. Data availability and accessibility: The manuscript reports the generation of OMIC-scale datasets, including scRNA-seq data, but does not provide accession numbers or a clear statement on public data deposition. In accordance with PLOS Pathogens data availability policies, the authors should specify the repository and corresponding accession number(s), or indicate that the data will be made publicly available upon publication.

Reviewer #2: Minor suggestions:

139, missing a comma after CA

160, 167, CFUs or CFU?

260, ISG, stimulated instead of “stimulate”

269, data revealed instead of “reveals”

528, fig1A, missing the median bar for C57BL/6 group.

The methodology does not contain sufficient details. For example, Listeria infection model is not found in the method.

Some description is not accurate. For example, line 174 “co-housing induced small changes to the composition of immune cells in the lung by day 14 post-infection”, changes were observed before infection.

Reviewer #3: As a complement to the last point above, the authors make an important finding that Lp exposure prior to Mtb infection limits spreading to the spleen : this is not commented that much and I actually believe that this finding is more relevant to the general role of innate immunity to the development of Mtb pathophysiology.

**********

PLOS authors have the option to publish the peer review history of their article (what does this mean?). If published, this will include your full peer review and any attached files.

If you choose “no”, your identity will remain anonymous but your review may still be made public.

**Do you want your identity to be public for this peer review?**  For information about this choice, including consent withdrawal, please see our Privacy Policy.

Reviewer #1: **Yes:**  Geanncarlo Lugo-Villarino

Reviewer #2: No

Reviewer #3: No

**Figure resubmission:**

While revising your submission, we strongly recommend that you use PLOS’s NAAS tool (https://ngplosjournals.pagemajik.ai/artanalysis) to test your figure files. NAAS can convert your figure files to the TIFF file type and meet basic requirements (such as print size, resolution), or provide you with a report on issues that do not meet our requirements and that NAAS cannot fix.

After uploading your figures to PLOS’s NAAS tool - https://ngplosjournals.pagemajik.ai/artanalysis, NAAS will process the files provided and display the results in the "Uploaded Files" section of the page as the processing is complete. If the uploaded figures meet our requirements (or NAAS is able to fix the files to meet our requirements), the figure will be marked as "fixed" above. If NAAS is unable to fix the files, a red "failed" label will appear above. When NAAS has confirmed that the figure files meet our requirements, please download the file via the download option, and include these NAAS processed figure files when submitting your revised manuscript.

**Reproducibility:**

To enhance the reproducibility of your results, we recommend that authors of applicable studies deposit laboratory protocols in protocols.io, where a protocol can be assigned its own identifier (DOI) such that it can be cited independently in the future. Additionally, PLOS ONE offers an option to publish peer-reviewed clinical study protocols. Read more information on sharing protocols at https://plos.org/protocols?utm_medium=editorial-email&utm_source=authorletters&utm_campaign=protocols

# Author comment: Author response to Decision Letter 1

**DOI:** 10.1371/journal.ppat.1014713.r002

19 Jun 2026

Attachment Submitted filename: ResponsetoReviews.pdf

# Peer review: Decision Letter 1

**DOI:** 10.1371/journal.ppat.1014713.r003

**Authors:** Thomas Guillard, Pierre Santucci

24 Jul 2026

PPATHOGENS-D-25-03003R1

Diverse infection models demonstrate robust resistance of Mycobacterium tuberculosis to innate immunity

PLOS Pathogens

Dear Dr. Vance,

Thank you again for your submission to PLOS Pathogens and the careful revision of your manuscript. After the re-assessment of your manuscript, the three experts have unanimously agreed that you addressed all the comments from the previous round of reviewing. Nevertheless, two experts have suggested to perform some text modification to discuss further these findings and concepts but also acknowledge some of the limitation of the study. You will see their comments below.

Should you address their comments and perform these minor modifications, we will be more than happy to officially accept your study for publication in PLOS Pathogens.

Please submit your revised manuscript by Sep 22 2026 11:59PM. If you will need more time than this to complete your revisions, please reply to this message or contact the journal office at plospathogens@plos.org. When you're ready to submit your revision, log on to https://www.editorialmanager.com/ppathogens/ and select the 'Submissions Needing Revision' folder to locate your manuscript file.

Please include the following items when submitting your revised manuscript:

* A letter that responds to each point raised by the editor and reviewer(s). You should upload this letter as a separate file labeled 'Response to Reviewers'. This file does not need to include responses to any formatting updates and technical items listed in the 'Journal Requirements' section below.

* A marked-up copy of your manuscript that highlights changes made to the original version. You should upload this as a separate file labeled 'Revised Manuscript with Track Changes'.

* An unmarked version of your revised paper without tracked changes. You should upload this as a separate file labeled 'Manuscript'.

If you would like to make changes to your financial disclosure, competing interests statement, or data availability statement, please make these updates within the submission form at the time of resubmission. Guidelines for resubmitting your figure files are available below the reviewer comments at the end of this letter.

As the corresponding author, your ORCID iD is verified in the submission system and will appear in the published article. PLOS supports the use of ORCID, and we encourage all coauthors to register for an ORCID iD and use it as well. Please encourage your coauthors to verify their ORCID iD within the submission system before final acceptance, as unverified ORCID iDs will not appear in the published article. Only the individual author can complete the verification step; PLOS staff cannot verify ORCID iDs on behalf of authors.

We look forward to receiving your revised manuscript.

Kind regards,

Pierre Santucci

Academic Editor

PLOS Pathogens

Thomas Guillard

Section Editor

PLOS Pathogens

Sumita Bhaduri-McIntosh

Editor-in-Chief

PLOS Pathogens

orcid.org/0000-0003-2946-9497

Michael Malim

Editor-in-Chief

PLOS Pathogens

orcid.org/0000-0002-7699-2064

**Reviewers' Comments:**

Reviewer's Responses to Questions

**Part I - Summary**

Please use this section to discuss strengths/weaknesses of study, novelty/significance, general execution and scholarship.

Reviewer #1: The authors have addressed all my major concerns conceptually and experimentally.

Reviewer #2: The conceptual advance of this study is that Mtb remains remarkably resistant to innate immune-mediated clearance in vivo, even under conditions in which innate immunity is broadly activated (Mtb infection itself, microbiota-educated hosts, or prior Legionella infection). This has important implications for how the field interprets macrophage antimicrobial assays. Numerous studies report altered bacterial growth in cultured macrophages following manipulation of specific innate pathways, yet it often remains unclear whether these effects translate into physiologically meaningful protection in vivo. I encourage the authors to emphasize this distinction more explicitly in the Abstract and Discussion. Specifically, the manuscript would benefit from a thorough discussion that cell-intrinsic restriction of Mtb growth in isolated macrophages does not necessarily predict host-level control of infection in the lung.

A related conceptual question also deserves discussion. If innate immunity has limited capacity to directly reduce Mtb bacterial burden, how do we interpret data reporting Mtb may evade or impair innate immunity, what's the benefit of doing so for the bacteria. And how should we interpret innate pathways that clearly influence disease susceptibility? Discussing this distinction would help place the present findings into the broader context of tuberculosis immunology.

Reviewer #3: The strength of this study is an original question : i.e. "does M tuberculosis does resist to innate immunity"? Yet, the weakness of the study remains in the models used that are very complicated and do vary along the study (for example the beginning is carried out with 1-3 ultra low dose infection and then the authors shift to 50 CFUs without further explanation). The readouts remain limited and there are still some potential pitfalls with i) the ULD model (and variable outcome of infection which could lead to misinterpretation) and ii) and interpretation of some immunological data. For example, the fact that Lp primed mice do control secondary infection better (see Figure 4A) could well be explained by Lp-specific onset of adaptive immunity making the sentence " This implies that priming with Lp strongly activates an anti-bacterial innate immune response" (line 208-209) an overstatement.

**********

**Part II – Major Issues: Key Experiments Required for Acceptance**

Please use this section to detail the key new experiments or modifications of existing experiments that should be absolutely required to validate study conclusions.

Generally, there should be no more than 3 such required experiments or major modifications for a "Major Revision" recommendation. If more than 3 experiments are necessary to validate the study conclusions, then you are encouraged to recommend "Reject".

Reviewer #1: The authors have addressed all my major issues, including key experiments, and the manuscript has improved dramatically in data interpretation, quality of results, and deeper discussion that accepts limitations and open possibilities. Therefore, this reviewer deems this manuscript fit for publication.

Reviewer #2: (No Response)

Reviewer #3: The revised version of the manuscript addresses most of my concerns even though to my opinion some data remain over interpreted (see above)

**********

**Part III – Minor Issues: Editorial and Data Presentation Modifications**

Please use this section for editorial suggestions as well as relatively minor modifications of existing data that would enhance clarity.

Reviewer #1: All minor issues have been addressed in the new version of the manuscript.

Reviewer #2: (No Response)

Reviewer #3: IN figure SFig4 it would be much more confortable to read the name of each mediator on top of the graph rather than looking for this information in the figure caption.

**********

PLOS authors have the option to publish the peer review history of their article (what does this mean?). If published, this will include your full peer review and any attached files.

If you choose “no”, your identity will remain anonymous but your review may still be made public.

**Do you want your identity to be public for this peer review?**  For information about this choice, including consent withdrawal, please see our Privacy Policy.

Reviewer #1: **Yes:**  Geanncarlo Lugo-Villarino

Reviewer #2: No

Reviewer #3: No

[NOTE: If reviewer comments were submitted as an attachment file, they will be attached to this email and accessible via the submission site. Please log into your account, locate the manuscript record, and check for the action link "View Attachments". If this link does not appear, there are no attachment files.]

**Figure resubmission:**

While revising your submission, we strongly recommend that you use PLOS’s NAAS tool (https://ngplosjournals.pagemajik.ai/artanalysis) to test your figure files. NAAS can convert your figure files to the TIFF file type and meet basic requirements (such as print size, resolution), or provide you with a report on issues that do not meet our requirements and that NAAS cannot fix.

After uploading your figures to PLOS’s NAAS tool - https://ngplosjournals.pagemajik.ai/artanalysis, NAAS will process the files provided and display the results in the "Uploaded Files" section of the page as the processing is complete. If the uploaded figures meet our requirements (or NAAS is able to fix the files to meet our requirements), the figure will be marked as "fixed" above. If NAAS is unable to fix the files, a red "failed" label will appear above. When NAAS has confirmed that the figure files meet our requirements, please download the file via the download option, and include these NAAS processed figure files when submitting your revised manuscript.

**Reproducibility:**

To enhance the reproducibility of your results, we recommend that authors of applicable studies deposit laboratory protocols in protocols.io, where a protocol can be assigned its own identifier (DOI) such that it can be cited independently in the future. Additionally, PLOS ONE offers an option to publish peer-reviewed clinical study protocols. Read more information on sharing protocols at https://plos.org/protocols?utm_medium=editorial-email&utm_source=authorletters&utm_campaign=protocols

# Author comment: Author response to Decision Letter 2

**DOI:** 10.1371/journal.ppat.1014713.r004

5 Oct 2026

Attachment Submitted filename: Response to Reviewers.pdf

# Editor report: Decision Letter 2

**DOI:** 10.1371/journal.ppat.1014713.r005

**Authors:** Thomas Guillard, Pierre Santucci

10 Oct 2026

Dear Dr. Vance,

We are pleased to inform that your manuscript, "Diverse infection models demonstrate robust resistance of Mycobacterium tuberculosis to innate immunity", has been editorially accepted for publication at PLOS Pathogens.

Before your manuscript can be formally accepted and sent to production, you will need to complete our formatting changes, which you will receive by email within a week. Please note that your manuscript will not be scheduled for publication until you have made the required changes.

IMPORTANT NOTES

(1) Copyediting and Proofreading: The corresponding author will receive a typeset proof for review, to ensure errors have not been introduced during production. Please review the PDF proof of your manuscript carefully, as this is the last chance to correct any errors. Please note that major changes, or those which affect the scientific understanding of the work, will likely cause delays to the publication date of your manuscript.

(2) Appropriate Figure Files: Please remove all name and figure # text from your figure files. Please also take this time to check that your figures are of high resolution, which will improve the readbility of your figures and help expedite your manuscript's publication. Please note that figures must have been originally created at 300dpi or higher. Do not manually increase the resolution of your files. For instructions on how to properly obtain high quality images, please review our Figure Guidelines, with examples at: http://journals.plos.org/plospathogens/s/figures.

We encourage you to use PACE (http://pace.apexcovantage.com/), a figure-checking tool which helps authors ensure all figures meet PLOS requirements so that figures can publish in the highest quality. PACE is a digital diagnostic and conversion tool for figure files. It will provide information about any failed check(s) and, if able, will automatically convert the figure into an acceptable file that passes quality checks.

To use PACE, you must first register as a user. Then, login and navigate to the UPLOAD tab, where you will find detailed instructions on how to use the tool.

(3) Striking Image: Please upload a striking still image to accompany your article if one is available (you can include a new image or an existing one from within your manuscript). Should your paper be accepted, this image will be considered for our monthly issue image and may also appear on our website to feature your article. Please upload this as a separate file, selecting "striking image" as the file type upon upload. Please also include a separate "Other" file with a caption, including credits and any potential copyright information. Please do not include the caption in the main article file. If your image is from someone other than yourself, please ensure that the artist has read and agreed to the terms and conditions of the Creative Commons Attribution License at http://journals.plos.org/plospathogens/s/content-license. Please note that PLOS cannot publish copyrighted images.

(4) Press Release or Related Media: If you or your institution will be preparing press materials for this manuscript, please inform our press team in advance at plospathogens@plos.org as soon as possible. We ask that you contact us within one week to plan ahead of our fast Production schedule. If you need to know your paper's publication date for related media purposes, you must coordinate with our press team, and your manuscript will remain under a strict press embargo until the publication date and time.

(5) Update your Profile Information: Now that your manuscript has been provisionally accepted, please log into Editorial Manager and update your profile, if needed. Go to http://www.editorialmanager.com/ppathogens, log in, and click on the "Update My Information" link at the top of the page. Please update your user information to ensure an efficient production and billing process.

(6) LaTeX users only: Our staff will ask you to upload a TEX file in addition to the PDF before the paper can be sent to typesetting, so please carefully review our Latex Guidelines http://journals.plos.org/plospathogens/s/latex in the meantime.

Thank you again for supporting Open Access publishing; we are looking forward to publishing your work in PLOS Pathogens.

Best regards,

Pierre Santucci

Academic Editor

PLOS Pathogens

Thomas Guillard

Section Editor

PLOS Pathogens

Dominique Soldati-Favre

Editor-in-Chief

PLOS Pathogens

***********************************************************

Dear Dr Vance,

Thanks for your time and careful revision of the manuscript. Congratulations on its acceptance, it is a beautiful piece of work.

Thank you again for submitting and contributing to PLOS Pathogens.

Sincerely,

Pierre

# Editor report: Acceptance letter

**DOI:** 10.1371/journal.ppat.1014713.r006

**Authors:** Thomas Guillard, Pierre Santucci

Dear Dr. Vance,

We are delighted to inform you that your manuscript, "Diverse infection models demonstrate robust resistance of Mycobacterium tuberculosis to innate immunity," has been formally accepted for publication in PLOS Pathogens.

We have now passed your article onto the PLOS Production Department who will complete the rest of the pre-publication process. All authors will receive a confirmation email upon publication.

Thank you again for supporting open-access publishing; we are looking forward to publishing your work in PLOS Pathogens.

Best regards,

Dominique Soldati-Favre

Editor-in-Chief

PLOS Pathogens
